# Exploring Adolescents’ Attitudes Toward Mental Health Apps: Concurrent Mixed Methods Study

**DOI:** 10.2196/50222

**Published:** 2024-01-15

**Authors:** Helene Høgsdal, Henriette Kyrrestad, Marte Rye, Sabine Kaiser

**Affiliations:** 1 Regional Centre for Child and Youth Mental Health and Child Welfare - North Faculty of Health Sciences Tromsø Norway

**Keywords:** mental health applications, mental health, adolescents, adolescent, youth, mobile health, app, apps, application, applications, opinion, opinions, cross sectional, survey, surveys, questionnaire

## Abstract

**Background:**

Adolescence is a critical time in which many psychological disorders develop. Mental health promotion is important, especially during this period. In recent years, an increasing number of mobile apps geared toward mental health promotion and preventing mental illness have been developed specifically for adolescents, with the goal of strengthening their mental health and well-being.

**Objective:**

This study aims to explore adolescents’ attitudes toward mental health apps, as well as the perceived usefulness of mental health apps.

**Methods:**

In this mixed methods study, a total of 183 adolescents (mean age 15.62, SD 3.21 years) answered a cross-sectional questionnaire, with 10 questions (eg, “What do you think about mental health apps in general?”). To complement the quantitative findings, individual interviews were conducted with 9 adolescents, during which they could elaborate on their opinions about mental health apps.

**Results:**

A total of 30% (56/183) of the adolescents in the quantitative study had used a mental health app. Over half of the respondents (77/126, 61.1%) reported that they would use a mental health app if they had a mental health problem as well as that they thought mental health apps were somewhat or very useful (114/183, 62.3%). Availability was the most frequently reported advantage of mental health apps (107/183, 58.8%). Possible associated costs of mental health apps were the most frequently mentioned barrier to their use (87/183, 47.5%). Findings from the interviews also pointed to the importance of the availability of mental health apps as well as their credibility and potential to provide adolescents with autonomy when seeking mental health advice and help.

**Conclusions:**

Overall, the results indicate that adolescents have a positive attitude toward and an interest in mental health apps. However, adolescents are also more or less unaware of such apps, which might be one reason why they are often not used. The findings of this study have important implications for future research on mental health apps and for developers of mental health apps that target young people. The insights gained from this study can inform the development of more effective mental health apps that better meet the needs and preferences of adolescents.

## Introduction

### Overview

The World Health Organization defines well-being as follows: “Well-being is a positive state experienced by individuals and societies. Similar to health, it is a resource for daily life and is determined by social, economic and environmental conditions” [1]. In today’s society, reference is often made to the importance of working to improve young people’s mental health and well-being as adolescence is a critical, transformative, and challenging time [2]. Research indicates that 10%-20% of adolescents worldwide experience mental health problems [3,4], and in approximately half of all mental disorders, the onset of symptoms occurs before the age of 15 years [5,6]. This illustrates that mental illness in adolescence can have negative effects also on adulthood [6,7]. Today, the need for mental health care and tools for young people is high, and it is argued that provided mental health services for adolescents are not sufficient to keep up with the growing demand [8]. It is therefore important to work toward developing targeted measures and evidence-based interventions that can help young people cope with stress and different challenges [2,9]. Furthermore, it is important to provide supporting tools and resources that can promote healthy habits and behaviors that contribute to overall well-being in adolescence.

In recent years, it has been suggested that mobile devices and the internet represent ideal tools to deliver such interventions to young people [10], and an increasing number of developers and researchers have been following this suggestion [11-14]. In a systematic review, Grist et al [13] highlight the importance of digital interventions developed for young people being of good quality. Furthermore, they point out that the development of digital products, intended for young people, should be designed in collaboration with young people, in order to develop customized and high-quality products for the target group. Thus, a broad understanding of what young people think about specific digital interventions can be beneficial for developing effective products suitable for the target group.

### Mental Health Mobile Apps

Mobile apps (ie, apps) are tools where users can receive digital health interventions or information, these health apps have the intention of improving the user’s overall health [12]. Along those same lines, mental health apps are designed to improve mental health and well-being among their users [14-16]. These apps can be designed to address specific mental health disorders [17,18], or they can be more general, focusing on promoting mental health and preventing mental distress through a variety of tools and resources such as emotional self-monitoring and coping strategies [19,20].

One important advantage of mental health apps is their availability [16]. Users can gain access to them anytime and anywhere with a smartphone or other mobile devices. This is particularly useful for individuals who may not have easy access to mental health services due to geographic barriers or for individuals who might benefit from receiving frequent reminders or immediate support [15,16]. Furthermore, mental health apps can reach larger segments of the population compared with in-person therapy, and, in some cases, they have shown promising cost-effectiveness [10,16].

### Adolescents’ Engagement With Mental Health Apps

Young people spend a considerable amount of time on their smartphones and use them for entertainment, social purposes, and to access information on different topics, including mental health [21-23]. There are several mental health apps available for adolescents, but as Grist et al [13] point out, the quality of the assessment on these apps is scarce. Research on mental health apps for adolescents indicates that young people are generally satisfied with the access to and ease of use of mental health apps [13,24]. Kenny et al [25] highlight in their study that adolescents prefer mental health apps to be safe, engaging, and easily accessible. The use of mental health apps can also allow people to maintain anonymity when seeking mental health advice or guidance, which is appreciated by young people, who often have a high threshold for help-seeking due to a desire for autonomy and the negative attitudes or stigma surrounding mental health services [26-28].

Nevertheless, the level of user engagement in existing mental health apps is relatively low or moderate and repeated long-time use of mental health apps after downloading them is rare [13,29,30]. This may indicate that despite the fact that young people find mental health apps appealing, it is not sufficient to ensure sufficient use over a long span of time. Regardless of the reasons, it is possible that low user engagement could decrease the overall effectiveness of mental health apps. Research on the effectiveness of mental health apps on adolescent mental health outcomes has revealed mixed results. Some research has failed to illustrate any effect [13,31,32], and others have indicated that mental health apps are promising and have the potential to provide improvement in mental health outcomes [14,33,34]. Yet, there is broad agreement that more research on the effectiveness of mental health apps is needed to fully understand their capacity to improve mental health outcomes [13,32,33]. In order to understand more about the effect mental health apps can have on young people’s mental health, it is also beneficial to examine what young people think about mental health apps and how they should appear in order to be effective for the target group.

### This Study

The aim of this study is to explore adolescents’ attitudes toward mental health apps, and their perceived usefulness, using both quantitative and qualitative data. While the quantitative study focuses more on the use and usefulness of these apps as well as on their perceived advantages and disadvantages, the qualitative study examines adolescents’ more general thoughts on mental health apps and how they may be of help to them. Together these data sets provide important insight for the development of mental health apps. The results can help to ensure more user-targeted products and more user engagement, which in turn can contribute to a greater effectiveness of mental health apps.

## Methods

### Overview

This study used a convergent, parallel QUAN + qual mixed methods design [35]. Quantitative and qualitative data were collected simultaneously, and the qualitative study was used to complement the results of the quantitative study. The quantitative data are based on a cross-sectional survey and the qualitative data are based on semistructured interviews. A total of 2 authors (HK and SK) analyzed the quantitative data, and the other 2 authors (HH and MR) analyzed the qualitative data. The results of the 2 data sets are integrated in the discussion.

### Quantitative Study

#### Participants and Procedure

Adolescents were invited to participate in the study during several events that took place in autumn 2022, including an innovation camp in Mosjøen (a municipality in Northern Norway), “The research days” at UiT The Arctic University of Norway, and the World Mental Health Day event organized by the municipality of Tromsø. To participate, adolescents were asked to scan a QR code, which led to a digital questionnaire [36]. Posters and flyers with the QR code were also distributed at various youth clubs and adolescent health centers in Northern Norway. The questionnaire was anonymous, and participation was voluntary. The final quantitative sample (N=183) consisted of 118 (64.5%) girls, 59 (32.2%) boys, and 6 (3.3%) adolescents that did not specify their gender. Participants were aged between 13 and 19 (mean 15.75, SD 1.65) years; 170 (92.9%) participants were Norwegian and 13 (7.1%) participants reported other nationalities (2.2% were Serbian, 1.1% were Swedish, and 3.8% did not specify their nationality).

#### Questionnaire

##### Overview

The questionnaire consisted of 10 questions. Information was collected on demographic characteristics like gender (boy, girl, and other), nationality (Norwegian, Serbian, Swedish, or other), and age (11 to 20 years). There were 5 or 6 questions about mental health apps that are described in the following and an open-ended question where the adolescents could give comments about the topic or the questionnaire in general.

##### Use of Mental Health Apps

Adolescents were asked 1 or 2 questions developed by Grist et al [37]: “Do you use (or have you used) any apps to help you with mental health problems?” with response options “yes” or “no.” Adolescents who answered “no” were asked a follow-up question: “If you had a mental health problem and there were apps available to help, would you use them?” with response options “yes” or “no.”

##### Perceived Usefulness of Mental Health Apps

Adolescents were asked: “What do you think of mental health apps in general?” with responses given on a 5-point Likert scale, ranging from (1) “not useful at all” to (5) “very useful.”

##### Advantages of Mental Health Apps

Adolescents were asked: “What do you think some of the advantages are of using an app for your mental health?” Adolescents were presented with 8 statements developed by Grist et al [37], such as “It is more private” and “I don’t have to talk to someone face to face.” One additional statement, “I can be anonymous” was added to the list by the authors. Adolescents could tick up to 3 statements with which they agreed the most*.*

##### Disadvantages of Mental Health Apps

Adolescents were asked: “What do you think some of the barriers are to using an app for your mental health?” Adolescents were presented with 9 statements developed by Grist et al [37], such as “I don’t trust apps,” “I am afraid someone will see the app on my phone,” and “It might cost money.” Adolescents could tick up to 3 statements with which they agreed the most.

##### Information About Mental Health

To examine where adolescents looked for information about mental health, they were asked: “Which media do you use to find information about mental health?” There were 7 response options as follows: Google, social media (eg, TikTok and Facebook), mental health apps, television, web-based newspapers, podcast or radio, and others, where adolescents could specify a different medium in an open-ended textbox. Adolescents were asked to tick all the media they used with no limitations.

#### Data Analytical Strategy

Data were analyzed with SPSS (version 29; IBM Corp). Descriptive statistics were calculated and included means, SDs, and frequency distributions. A multiple linear regression analysis was calculated to predict adolescents perceived usefulness of mental health apps, with gender (1=boy, 2=girl), age (11-20 years), and previous use of mental health apps (1=yes, 2=no) as predictors. A significance level of less than .05 was applied.

### Qualitative Study

#### Participants and Procedure

Adolescents who answered the questionnaire were invited to contact the researchers if they wanted to participate in an interview and share their thoughts and opinions about mental health apps. A total of 9 adolescents (all Norwegian) chose to be interviewed (Table 1); 7 elected to be interviewed remotely (via telephone, Microsoft Teams, or Zoom) and 2 elected to be interviewed in person. Before each interview, the adolescents were informed that they were expected to talk about mental health apps, which were defined as mobile apps “developed with the thought of helping people manage their own mental health and wellness” [16]. All interviews were audio recorded on an Apple iPad with the app Diktafon [38], encrypted, and sent directly to Nettskjema [36] before they were transcribed. Two adolescents requested to be interviewed together, hence, 1 interview was conducted with 2 adolescents present (informant 3 and informant 4). These adolescents were considered as individual informants despite being from the same interview.

**Table 1:** Overview of informants and their experience with mental health apps.

Informant	Gender	Age (years)	Experience with mental health apps
Informant 1	Girl	15	No
Informant 2	Boy	16	No
Informant 3	Girl	15	Yes
Informant 4	Boy	16	No
Informant 5	Girl	14	No
Informant 6	Girl	17	No
Informant 7	Girl	17	Yes
Informant 8	Boy	16	Yes
Informant 9	Girl	14	Yes

#### Interviews

All interviews were conducted in Norwegian. The interviewer followed a semistructured interview guide, however, the adolescents were encouraged to talk freely without too much interruption from the interviewer. There were three key questions in the interview guide: (1) “What are your thoughts on mental health applications that are aimed at adolescents?” (2) “What do you think mental health applications can do with regard to adolescents’ knowledge about mental health?” (3) “How do you think mental health applications may be of help to young people?” The interviewer asked follow-up questions when it was natural to go more in-depth on the information the adolescent provided. The interviews varied in duration from 15 to 30 minutes.

#### Analytic Strategies

The qualitative data were analyzed using thematic analysis inspired by Braun and Clarke [39]. Our analysis was based on a constructionist epistemological position, assuming knowledge is socially constructed and developed through communication and interactions between people [40]. Audio recordings were transcribed by the first author, and transcripts were imported into NVivo [41] to support the organization and coding of the data. Transcripts were read carefully by the first and third authors to identify meaning and patterns in the data. Initial codes were created for the data material, which were then sorted into potential themes. The themes were reviewed and defined in order to identify the essence of each one and the relationship between them. All the codes, themes, and presented quotes are based on data from the Norwegian language. The translation was done using a contextualized hermeneutic approach to translation [42] where the data are presented as close to the original context as possible, hence the quotations in particular are directly translated from Norwegian to English.

### Ethical Considerations

The questionnaire in the quantitative study was anonymous and no personally identifiable information was collected. To complete the questionnaire, adolescents had to scan the QR code of their own volition. Moreover, before they could access the questionnaire, the adolescents were presented with information about the study and they were told that they agreed to participate by answering the questions. The adolescents were also informed that they could stop answering the questionnaire at any time without consequences.

The qualitative study was evaluated and approved by the Norwegian Center for Research Data (reference 631424). Adolescents of 16 years or older could consent to take part in the interview themselves, while active parental consent was required from adolescents younger than 16 years of age. Consent was retrieved via a digital consent form in Nettskjema [36]. All interview informants received a cinema gift card with a value of NOK 150 (approximately US $15) as compensation.

## Results

### Quantitative Study

Of the 183 adolescents who completed the web-based questionnaire, 56 (30.6%) adolescents had used a mental health app. Among adolescents who had not used a mental health app (126/183, 68.9%), approximately half (77/126, 61.6%) said they would use one if they had a mental health problem and there were apps available to help, while 48 (38.4%) adolescents said they would not. When asked “What do you think about mental health apps in general?” 25 (13.7%) adolescents answered that they were not or not very useful, 44 (24.0%) adolescents answered “neither”, and 114 (62.3%) adolescents found them somewhat or very useful (mean 3.6, SD 1.1). The regression analysis to predict the perceived usefulness of mental health apps, identified only gender as a significant predictor (β=.35; *P*<.001) indicating that girls perceived mental health apps as more useful than boys.

The most frequently reported advantages of using a mental health app were as follows: “It will always be there when I need it” (107/183, 58.8%), “I don’t have to talk to someone face to face” (83/183, 45.4%), “It is more private” (75/183; 41.0%), and “I can be anonymous” (72/183, 39.3%; Table 2). The most frequently reported barriers were as follows: “It might cost money” (87/183, 47.5%), “I don’t know whether the information in them is accurate or true” (82/183, 44.8%), and “I am afraid someone will see the app on my phone” (78/183, 42.6%; Table 3). The most frequently cited media that adolescents used to find information about mental health were by far “Google” (151/183, 82.5%) and “Social media (for example Facebook, Instagram, TikTok)” (90/183, 49.2%; Table 4).

**Table 2:** Advantages of using mental health apps (N=183).

Statement	Values, n (%)
It is more private	75 (41.0)
I don’t have to talk to someone face to face	83 (45.4)
It will always be there when I need it	107 (58.8)
I don’t have to wait to get information	45 (24.6)
I can get support and information whenever I need it	51 (27.9)
I don’t have to write things like my mood down on paper	13 (7.1)
It is personal to me	12 (6.6)
I can be anonymous	72 (39.3)
Other	6 (3.3)

**Table 3:** Barriers to using mental health apps (N=183).

Statement	Values, n (%)
I don’t trust apps	49 (26.8)
I don’t know whether the information in them is accurate or true	82 (44.8)
I would prefer to speak to someone face to face	46 (25.1)
I don’t think apps can help me	36 (19.7)
I am afraid someone will see the app on my phone	78 (42.6)
It might cost money	87 (47.5)
Other	17 (9.3)

**Table 4:** Most often used media to find information about mental health (N=183).

Media	Values, n (%)
Google	151 (82.5)
Social media (eg, Facebook, Instagram, TikTok)	90 (49.2)
Mental health mobile apps	23 (12.6)
Television	28 (15.3)
Web-based newspapers	8 (4.4)
Podcast or radio	26 (14.2)
Other	12 (6.6)

### Qualitative Study

#### Theme 1: Accessibility—The Significance of Approachable Mental Health Apps

A central theme that arose from the interviews, was the experienced and perceived accessibility of mental health apps. The adolescents highlighted that mental health apps were a readily accessible alternative to use in order to find information about health care as well as to get help. Further, they pointed out that mental health apps could be a more accessible helping tool than person-to-person offers. In addition, the adolescents talked about accessibility in terms of knowing about mental health apps’ existence, how appealing they are, and potential costs related to using them.

Many of the young people pointed out that they had neither heard of nor used mental health apps.

I don't really know about any health-promoting apps that young people use

informant 6

Some adolescents reflected on reasons why they had not heard of them. For instance 1 adolescent stated that her lack of knowledge of mental health apps may be attributable to marketing campaigns that do not target young people directly or correctly.

I don't think, for example, that the makers of such apps have managed to reach out to young people [...]. I don't know if I'm perhaps in the wrong target group or something, but at least it hasn't really caught on yet, so to speak

informant 1

The adolescents also pointed out that the information in the mental health apps must be easy to understand to be appealing to them. The adolescents stated that information found on internet might be challenging to comprehend, due to for example the use of specialized terminology. Therefore, they highlighted that it is important that the information provided in mental health apps is specific to the topic that they want to learn more about.

I think that can be good, if you have a good app where the information is simply explained and that you understand it quite easily, then I think it can be used a lot

informant 9

An app should be a place where people can read about exactly what they need and not so much medical stuff

informant 1

The importance of free mental health apps was frequently mentioned in all interviews; adolescents stressed that apps should be equally accessible to everyone regardless of their economic situation. Further, some adolescents stated that charging a fee would weaken the credibility of such apps and call into question the developers’ intentions.

I've been on an app where it was obvious that they were going to make money off it. And then the information and everything seemed a bit wrong. Suddenly, I had to pay for a membership in order to continue to use the app; then they lost me

informant 3

#### Theme 2: Trustworthiness—The Significance of Credible Mental Health Apps

Most adolescents stated that it is important that app developers are credible and that the information that is provided in apps comes from reliable sources.

It is important that young people find a reliable source. That it's not just random journalists, or someone who is just trying to get clicks, but that it comes from someone who actually knows something about it. So that you know you are getting the right information

informant 2

Some adolescents also assumed that it would be easier to judge whether the information on a mental health app is correct, as compared with determining the accuracy of the information on the internet. One adolescent stated that apps developed by established, credible sources would be easier to trust than those created by unknown sources.

I think it would be very easy to just take in everything that's written there [in a mental health app]. Even the bad stuff. That is why I think that such an app should somehow be under Ung.no [a public information channel for young people in Norway] or something, some reliable sources

informant 5

#### Theme 3: Autonomy—Mental Health Apps Can Help Adolescents Help Themselves

Several adolescents stated that self-determination was important to them, so they would appreciate an app, as it would allow them to manage their own mental health and difficulties without interference from adults.

In a way, you want to try to help yourself before it goes so far that you are dependent on others to help you

informant 1

An app should contain information about what we can do ourselves to get better and where we can ask for help. Sometimes you may just need to get guidelines on how to do it yourself

informant 8

Further, several adolescents pointed out that an app can be used to ask for help if one does not *want* to contact in-person services.

I think it's good that there are apps like that, that you don't have to go and talk to someone, but that you can have an app on your phone that you have all the time

informant 9

Most adolescents stated that the apps should include a way to communicate with someone.

If it [an app] can be made so that you can communicate with someone, then perhaps the threshold [of asking for help] will be even lower

informant 1

Mm, it would have been good if the app contained something like that, a way to talk to a person online

informant 2

Adolescents were also interested in interacting within the app in ways that did not involve another person. Some suggested that the app could contain some predefined questions that could be used to give the user a more personalized experience, or that the app could refer them to the right professional.

Several adolescents also pointed out that contacting in-person services can trigger undesirable actions, which might make it easier to trust an app.

And you are probably afraid that it will somehow..., you want to deal with it, but at the same time you are afraid that, for example, a nurse or someone will tell someone else and that there will be a lot of actions at school, and then everyone will know about it

informant 1

Finally, a frequently mentioned topic in the interviews was the importance of being anonymous in the apps. Adolescents cited the value they placed on anonymity both if they needed information and if they needed actual help with their mental health, as this could make it easier for the adolescents to seek help.

It is important that you can remain anonymous, because not everyone likes to talk about such things if you are recognizable

informant 6

## Discussion

### Principal Findings

In this study, we examined adolescents’ attitudes and general thoughts toward mental health apps using a concurrent, mixed methods approach. This research is an important contribution to understanding what young people think about mental health-promoting mobile apps, as well as their thoughts on what they should include and how they should appear.

Overall, the adolescents expressed positive attitudes toward mental health apps, even though few had experience with using them. A large proportion perceived such apps as useful tools that can help them cope with normal stresses of life, which is important in order to promote well-being and prevent mental health problems [43,44]. In the quantitative section of the study, availability was the most chosen benefit of using mental health apps. This is not surprising as adolescents in Norway spend a lot of time on their phones and on social media [21]. Privacy, as well as the possibility of remaining anonymous, was the subsequent most chosen advantage. These results are in accordance with those reported in previous literature [16,25,37]. Further, in the interviews, adolescents expressed that mental health apps can be of help at any time of the day, also when in-person services are not available. However, the adolescents expressed, that for mental health apps to be accessible, they need to also be easily understandable and have no costs attached.

It is well-known that adolescents tend to be reluctant to seek help, despite the importance of obtaining help and support for improved well-being [45-47]. In this study, adolescents expressed that mental health apps can lower adolescents’ threshold for asking for help, by always being available and by offering anonymity in the help-seeking process. Several interviewees also highlighted the empowering potential of mental health apps. The adolescents valued how an app could provide them with the ability to help themselves without interference from others. Meeting young people’s need for autonomy is important to ensure that they ask for help when they need it [28]. The findings from this study concur with previous findings, which suggested that adolescents value the autonomy that mental health apps provide [48].

Further, the adolescents emphasized that mental health apps should provide an anonymous way for them to talk about their problems, with either a web-based assistant or a real person. In addition, some adolescents reported fear of someone else seeing a mental health app installed on their phone as a deterrent to using such apps. Anonymity is often mentioned as an important reason why adolescents use web-based services when searching for help or advice regarding mental health [16,49], our findings support that adolescents view anonymity as an advantage of using mental health apps, which agrees with previous findings [16,25].

Another important aspect adolescents highlighted was the difficulty they might have in judging whether the information contained in a mental health app was accurate. Most adolescents reported that they used the internet (ie, Google and social media) to search for mental health-related information. The adolescents stated that mental health apps should be developed by credible organizations or individuals that adolescents already trust. Adolescents also stated that, the information presented in the app should be clear and concise in order to be engaging for them. Previous research have also shown the importance of content and appearance to ensure engaging mental health apps [25,50].

Although adolescents generally have a positive attitude toward the increased development and use of mental health apps, few of the adolescents in our study sample were familiar with such apps. These results are in line with previous research which indicates that adolescents are not highly engaged in mental health apps [13,29]. Indeed, some adolescents believed that they had not heard of such apps due to poor marketing strategies. They stated that, if the apps were meant to target adolescents, then they should be marketed to young people directly. However, there are currently strict rules regarding advertising to children and adolescents in Norway [51], which may pose challenges in devising effective marketing strategies to reach adolescents.

### Strengths and Limitations

By including both quantitative and qualitative data, this study provides valuable knowledge and insight into adolescents’ perspectives and opinions on mental health apps. However, there are several limitations that must be taken into consideration. In the quantitative study, the sample size was relatively small and not representative. One should therefore be careful about drawing general conclusions from the findings. However, the results can be an important contribution to the field of research on what adolescents think about mental health apps. Moreover, the interviews were of short duration, which may have influenced the depth of the answers. However, the adolescents were encouraged to speak freely, and they were not interrupted until they had concluded. Follow-up questions were asked when they brought up topics that the interviewer found interesting or considered meaningful for the adolescent to elaborate on. Furthermore, because the qualitative study was intended to complement the quantitative study, we perceive the length and depth of the interviews as sufficient.

One interview was conducted with 2 adolescents together, based on the participants’ own desires. Since they stated it would increase their sense of security during the interviews, the request was accepted. However, we cannot exclude the possibility that the presence of another adolescent influenced the answers the adolescents provided.

### Conclusions

Mental health apps can be a useful resource for adolescents, and several apps geared toward adolescents have been developed. Our findings show that there is a lack of knowledge about the existence of mental health apps among adolescents. A large proportion of adolescents expressed that they would use these apps if they knew they were available. This study shows that apps directed toward adolescents should be easily accessible, free of charge, and provide easily understandable information. Adolescents also emphasized the significance that apps should be developed by credible sources or institutions, offer a choice between human and web-based support, and enable users to remain anonymous while seeking help. Future development of mental health apps should take these considerations into account.

## Data Availability

The data sets generated or analyzed during this study are available from the corresponding author on reasonable request.
